# Gibberellin-Induced Transcription Factor *Sm*MYB71 Negatively Regulates Salvianolic Acid Biosynthesis in *Salvia miltiorrhiza*

**DOI:** 10.3390/molecules29245892

**Published:** 2024-12-13

**Authors:** Cuicui Han, Xingwen Dong, Xiaowen Xing, Yun Wang, Xiaobing Feng, Wenjuan Sang, Yifei Feng, Luyao Yu, Mengxuan Chen, Hongyuan Hao, Taohong Huang, Bailin Li, Wenhui Wu, Zheng Zhou, Ying He

**Affiliations:** 1 College of Food Science and Technology, Shanghai Ocean University, Shanghai 201306, China; hancuicui1910@163.com (C.H.); blli@shou.edu.cn (B.L.); whwu@shou.edu.cn (W.W.); 2 Navy Special Medical Centre, Second Military Medical University, Shanghai 200433, China; 3 Biomedical Innovation R&D Center, School of Medicine, Shanghai University, Shanghai 200444, China; 4 Shanghai Analytical Applications Center, Shimadzu (China) Co., Ltd., Shanghai 200233, China; 5 National Key Laboratory of Immunity and Inflammation, Naval Medical University, Shanghai 200233, China

**Keywords:** salvianolic acid, *Sm*MYB71, gibberellins, *Sm*ERF115, biosynthesis

## Abstract

*Salvia miltiorrhiza*, the valuable traditional Chinese medicinal plant, has been used in clinics for thousands of years. The water-soluble salvianolic acid compounds are bioactive substances used in treating many diseases. Gibberellins (GAs) are growth-promoting phytohormones that regulate plant growth and development. Previous studies have demonstrated that GAs can promote salvianolic acid accumulation in *S. miltiorrhiza*; however, the underlying mechanism requires further investigation. Here, we identified a GA-induced R2R3MYB transcription factor (TF), *Sm*MYB71, from a transcriptome library of GA-treated *S. miltiorrhiza*. *Sm*MYB71 was highly expressed in the root of *S. miltiorrhiza* and localized to the nucleus. *Sm*MYB71-knockout hairy roots showed higher salvianolic acid accumulation compared to wild lines. Overexpressing *Sm*MYB71 in *S. miltiorrhiza* hairy roots significantly decreased the content of salvianolic acid by downregulating key salvianolic acid biosynthesis enzymes such as *Sm*RAS and *Sm*CYP98A14. The GCC box in the promoter of *Sm*MYB71 can bind with *Sm*ERF115, suggesting that *Sm*MYB71 is regulated by *Sm*ERF115 in salvianolic acid biosynthesis. These findings demonstrate a novel regulatory role of *Sm*MYB71 in GA-mediated phenolic acid biosynthesis. With the development of CRISPR/Cas9-based genome editing technology, the *Sm*MYB71 regulation mechanism of salvianolic acid biosynthesis provides a potential target gene for metabolic engineering to increase the quality of *S. miltiorrhiza*.

## 1. Introduction

**Figure 1:**
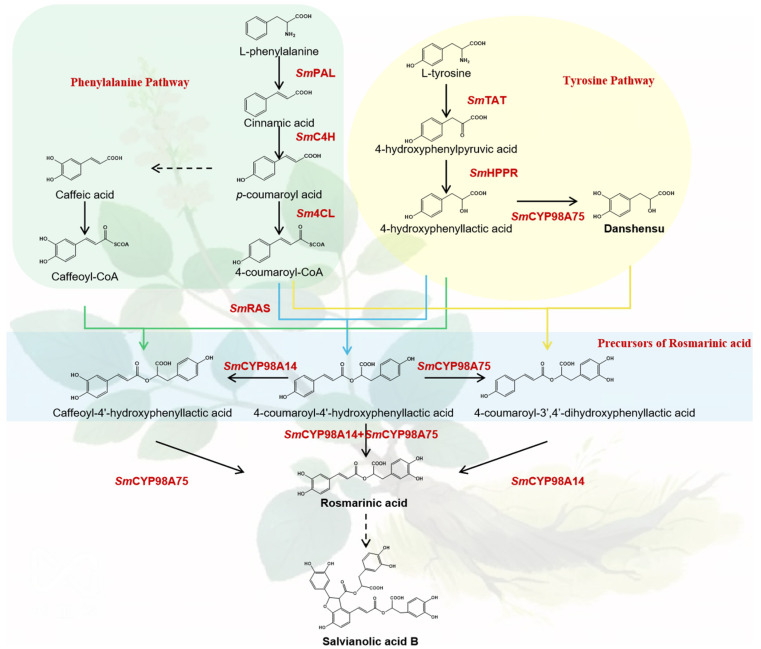
Biosynthetic pathways of salvianolic acid synthesis in *S. miltiorrhiza*. PAL: L-phenylalanine ammonia-lyase; C4H: cinnamate 4-hydroxylase; 4CL: 4-coumaric acid coenzyme A ligase; TAT: L-tyrosine aminotransferase; HPPR: hydroxyphenylpyruvic acid reductase; RAS: rosmarinic acid synthase; CYP98A: cytochrome P450 98A.

*Salvia miltiorrhiza* is atraditional Chinese medicinal herb, used for treatment of cardiovascular and cerebrovascular diseases for thousands of years. The major bioactive salvianolic acids, including danshensu (DSS), caffeic acid (CA), rosmarinic acid (RA) and salvianolic acid B (SAB) are widely used in anti-inflammatory, anti-antioxidant anti-microbial, as well as anti-tumor treatments [1,2]. The salvianolic acid biosynthesis pathway is a non-linear pathway which contains phenylpropanoid- and tyrosine-based pathways. The acyl donors (4-coumaroyl-CoA and caffeol-CoA) from phenylpropanoid and acyl acceptors (4-hydroxyphenyllactic acid and DSS) from the tyrosine pathway are catalyzed by the rosmarinic acid synthase (*Sm*RAS) and CYP98A (Cytochrome P450 98A) family of enzymes to synthesize RA. The laccase family was speculated to dimerize RA to generate SAB (Figure 1). The regulation mechanism of salvianolic acid generation also garnered researchers’ attention and transcription factors (TFs), as dominant regulation genes, play significant roles in the biosynthesis of salvianolic acid.

MYB TFs are a large plant TF family and are generally divided into four subfamilies, 1R-MYB, R2R3-MYB, 3R-MYB, and 4R-MYB, based on the location and number of conserved MYB-domain adjacent repeats. R2R3-MYBs are the biggest subfamily among MYB TFs and are involved in plant growth and development, secondary metabolism, and biotic and abiotic stress responses, as well as cell-fate decision processes. For instance, an R2R3-MYB TF *Ps*FLP regulates the symmetric division of guard mother cells during stomatal development in *Pisum sativum* [3]. In *Populus*, *Pt*MYB6 promotes flavonoid biosynthesis but inhibits secondary cell wall formation. Overexpression of *Pt*MYB6 in transgenic poplar lines upregulated flavonoid biosynthesis gene expression, resulting in significantly increased accumulation of anthocyanin and proanthocyanidins, accompanied by reduced secondary cell wall deposition [4]. *Ta*MYB344 was identified from mining of the abiotic and biotic stress treatment RNA-seq database of *Triticum aestivum*. Overexpression of *Ta*MYB344 in tobacco (*Nicotiana tabacum*) plants enhanced drought, heat, and salt stress tolerance [5].

In *S. miltiorrhiza*, a total of 291 MYB TFs were identified and several of these TFs have been reported to be related to salvianolic acid biosynthesis [6]. For instance, *Sm*MYB4 negatively regulated key enzymes in salvianolic acid and tanshinone biosynthesis, and the content of salvianolic acid and tanshinones decreased in *Sm*MYB4-overexpression lines but increased in *Sm*MYB4-repression lines [7]. The transcription complex of *Sm*MYB111-*Sm*TTG1-*Sm*bHLH51 is the first report of a potential ternary complex in *S. miltiorrhiza*, involved in production of salvianolic acid. *Sm*MYB111 positively regulated salvianolic acid biosynthesis and interacted with *Sm*TTG1 and *Sm*bHLH51 [8]. Overexpressed *Sm*MYB52 significantly inhibited root growth and indole-3-acetic acid accumulation, whereas it activated phenolic acid biosynthesis, which indicated that *Sm*MYB TF was simultaneously involved in root growth and development as well as the accumulation of salvianolic acid [9]. These studies uncovered the relationship of *Sm*MYB TF’s in regulating salvianolic acid biosynthesis.

Phytohormones, such as MeJA, abscisic acid (ABA), gibberellins (GA), and ethylene, etc., play crucial roles as signaling molecules in various processes of plant growth and development, as well as in the regulation of secondary metabolism. Previous studies have shown that MeJA, GA, and ABA are all effective in inducing the production of salvianolic acid and increasing the expression level of key enzymes in salvianolic acid biosynthesis [10,11]. *Sm*MYB1 and *Sm*MYB2 are MeJA (Jasmonic acid methyl ester)-responsive R2R3-MYB TFs, which were characterized from the MeJA-induced transcriptome database of *S. miltiorrhiza*; they dramatically promoted salvianolic acid accumulation and upregulated the *SmCYP98A14* expression level in the salvianolic acid biosynthesis pathway after overexpression [7,12]. ABA also plays essential roles in plant growth and development, especially in environmental stress responses including responses to drought, salinity, and UV stress. Several TFs in *S. miltiorrhiza* have been verified to regulate the accumulation of salvianolic acid and tanshinones. Overexpression of ABA-responsive TF *Sm*bZIP1 positively promoted salvianolic acid content by enhancing the expression of key salvianolic acid biosynthesis enzymes such as C4H1 and negatively reduced tanshinone accumulation by suppressing geranylgeranyl diphosphate synthase (GGPPS) [13]. *Sm*WD40-170, a WD40 TF family member, which is regulated by ABA and H_2_O_2_, was identified as a critical drought response gene related to NO and ROS signaling [14]. These findings suggest that phytohormones play a crucial role in salvianolic acid biosynthesis.

Most phytohormone studies primarily focus on the regulatory mechanisms of TFs mediated by MeJA and ABA, while the GA TF network has received comparatively less attention in *S. miltiorrhiza*. GAs are diterpene hormones that are generated via complex pathways and control plant growth and development processes, including seed germination, stem elongation, leaf expansion, and flower development. There are more than 130 GAs that have been characterized in different plant species, yet only a few, such as GA_1_, GA_3_, GA_4_, and GA_7_. As for studies of GAs in *S. miltiorrhiza*, 35 *Sm*GRAS TFs have been identified and divided into 10 subfamilies [11]. Overexpression of *Sm*GRAS1 and *Sm*GRAS2 upregulated the tanshinone content and downregulated GAs, phenolic acid content, and root biomass. Antisense expression of *Sm*GRAS1 and *Sm*GRAS2 reduced the tanshinone accumulation and increased the GAs, phenolic acid content, and root biomass. The study identified the relative expression levels of genes in the GA biosynthesis pathway. In the *Sm*GRAS1/2-overexpression lines, most of the downstream genes in GA biosynthesis were inhibited, except for GA20ox2/6. This observation indicated that *Sm*GRAS1 and *Sm*GRAS2 modulated root development by influencing GA biosynthesis [15]. Considering that the MYB family is the largest TF family in plants, GA-induced MYB TFs have been reported in various plant species. The *At*MYB12 TF, a member of subgroup 7 R2R3-MYB TF, interacted with DELLAs to enhance the binding affinity of MYB to promoter regions of key genes involved in flavonol biosynthesis, consequently increasing their transcription levels. Genetic experiments demonstrated that loss-of-function mutations in MYB12, primarily expressed in roots, partly rescued the short-root phenotype of the GA-deficient mutant ga1-3 by enlarging the root meristem and mature cell size. Consistently, the application of the flavonol quercetin externally restored the root meristem size of MYB12 ga1-3 mutants to that of ga1-3 mutants. These findings shed light on a molecular mechanism where GAs facilitate root growth by directly suppressing flavonol biosynthesis [16]. Overexpression of the *MYBH* gene significantly promoted the growth of the hypocotyls in *Arabidopsis* seedlings, while gibberellin synthesis inhibitors markedly impeded this growth [17]. These findings confirmed the roles of MYB TFs in plant growth and secondary metabolism. However, reports on GA-responsive MYB TFs concerning *S. miltiorrhiza* are limited; therefore, it is essential to focus on their role in GA regulation.

CRISPR/Cas9 technology, often referred to as “genetic scissors”, is heralded as a revolutionary technology with significant potential for creating designer crops. This innovation introduces precise and targeted modifications to the genome, offering a pathway to achieve global food security amid climate change and a rising population. In contrast, traditional genetic engineering relies on the random and unpredictable insertion of isolated genes or foreign DNA elements into the plant genome. Conversely, CRISPR-Cas-based gene editing allows for the introduction of new traits by precisely altering existing genes without necessitating the insertion of foreign DNA from different species. Utilizing CRISPR/Cas9 to enhance the content of active medicinal ingredients represents a promising strategy for improving the quality of medicinal plants. Therefore, the knockout of negatively regulating TFs may effectively increase the accumulation of bioactive ingredients. Here, we discovered an R2R3-MYB TF *Sm*MYB71, which was induced by GA_3_ and negatively regulated salvianolic acid biosynthesis. Knockout of *Sm*MYB71 resulted in higher salvianolic acid accumulation in transgenic hairy roots compared to wild-type (WT) lines. Overexpressing *Sm*MYB71 in *S. miltiorrhiza* hairy roots significantly decreased the content of salvianolic acid via downregulating salvianolic acid biosynthesis key enzymes. In addition, electrophoretic mobility shift assay (EMSA) experiments confirmed the interaction between *Sm*ERF115 and *Sm*MYB71, which suppressed the expression of *Sm*MYB71 and ultimately promoted the biosynthesis of salvianolic acid. These findings demonstrated a novel regulatory role of *Sm*MYB71 in GA-mediated salvianolic acid biosynthesis, suggested *Sm*MYB71 as a potential target gene for metabolic engineering, and elucidated the regulatory network of salvianolic acid.

## 2. Results

### 2.1. GA_3_ Negatively Regulated SmMYB71 Gene Expression

**Figure 2:**
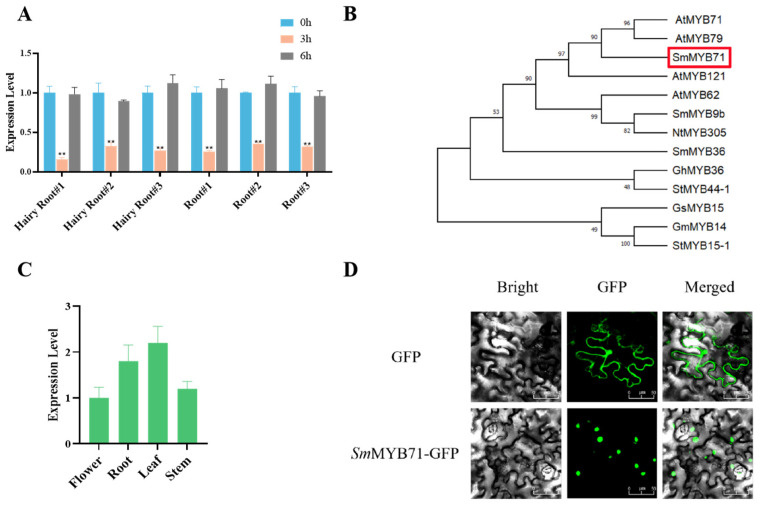
(**A**) Expression level of the *SmMYB71* gene in *S. miltiorrhiza* and hairy roots following GA treatment. Following GA_3_ stimulation, the expression level of the *SmMYB71* gene demonstrated a significant initial decrease. Three hours post-treatment, the expression reached its lowest point. However, after six hours, the expression level gradually returned to baseline (** *p* < 0.01). (**B**) Evolutionary analysis of *MYB* genes from various plant species. The protein accession numbers are as follows: *At*MYB71(GenBank:NP_189074.1), *At*MYB79(GenBank:NP_193084.1), *At*MYB121(GenBank:NP_189640.1), *At*MYB62(GenBank:NP_176999.1), *Sm*MYB9b(GenBank:AGG09670.1), *Sm*MYB36(GenBank:AGN52060.1), *Nt*MYB305(GenBank:EU111679.1), *Gh*MYB36(GenBank:ASH96785.1), *St*MYB44-1(GenBank:QCH00894.1), *St*MYB15-1(GenBank:QCH00896.1), *Gs*MYB15(GenBan-k:AZZ09187.1), *Gm*MYB14(GenBank:AHC55374.1). The red box indicates *Sm*MYB71. (**C**) Expression of the *SmMYB71* gene in the roots, stems, leaves, and flowers of *S. miltiorrhiza*. The highest content of *SmMYB71* was found in the leaves of *S. miltiorrhiza*, while the lowest content was observed in the stems. (**D**) Determination of the subcellular localization of *SmMYB71* through the transient expression of GFP-tagged proteins in the leaves of *Nicotiana benthamiana*. Scale bars represent 25 μm.

We utilized a previously established transcriptome database of gibberellin (GA)-induced hairy roots of *S. miltiorrhiza* (0 h, untreated control; treated for 2 h with GAs), which is available in the Sequence Read Archive (SRA) of the National Center for Biotechnology Information (NCBI) under accession number PRJNA663993, to identify R2R3-MYB TFs associated with salvianolic acid biosynthesis in *S. miltiorrhiza* [11]. The expression levels of several R2R3-MYB TF-encoding genes within the transcriptome database were found to be downregulated dramatically in response to GA treatment. These TFs are promising negative regulators of salvianolic acid biosynthesis. Among these genes, we selected *SmMYB71* (GenBank accession number AGN52058.1) for functional analysis because its expression profile inversely correlates with that of the salvianolic acid biosynthesis pathway genes *Sm4CL*, *SmRAS*, and *SmCYP98A14* under GA treatment. To further validate the response of *SmMYB71* to GAs, we treated two-month-old *S. miltiorrhiza* plants and hairy roots with 100 μM GA_3_ solution. The qRT-PCR technique was employed to assess the expression levels of the *SmMYB71* gene in the treated leaves and roots of *S. miltiorrhiza*. The results indicate that *Sm*MYB71 exhibits a dramatic response to induction by GA_3_. Following GA_3_ stimulation, the expression level of the *SmMYB71* gene demonstrates a significant initial decrease. Three hours post-treatment, the expression reaches its lowest point. However, after six hours, the expression level gradually returns to baseline (Figure 2A).

### 2.2. Isolation and Characterization of SmMYB71

The ORF of the *SmMYB71* gene comprises 702 bp, resulting in the translation of a protein consisting of 233 amino acids. To further examine the evolutionary relationships among *Sm*MYB71 and other MYB proteins, we constructed a phylogenetic tree that included representatives from tobacco, cotton, soybean, and potato. The results indicated that *Sm*MYB71 exhibited the highest similarity to *At*MYB71 and *At*MYB79 from *Arabidopsis thaliana*, with similarity percentages of 57% and 56%, respectively (Figure 2B).

### 2.3. Expression Analysis of SmMYB71

To investigate the expression pattern of the *SmMYB71* gene, we assessed its expression in the roots, stems, leaves, and flowers of *S. miltiorrhiza* using qRT-PCR. The results indicated that the expression level of *SmMYB71* was highest in the leaves and lowest in the stems (Figure 2C). Furthermore, to confirm the cellular localization of *SmMYB71*, subcellular localization experiments were conducted, during which the GFP-*SmMYB71* fusion vector was transiently expressed in *N. benthamiana* (Figure 2D). The results revealed that *SmMYB71* localized to the nucleus, the primary site of transcription, aligning with its function as a TF that regulates biological processes. Moreover, salvianolic acids are known to accumulate in the leaves and roots, suggesting that the high expression of *SmMYB71* in both tissues may influence metabolite accumulation.

### 2.4. SmMYB71 Negatively Regulated Salvianolic Acid Biosynthesis

To investigate the role of *Sm*MYB71 in salvianolic acid biosynthesis, we constructed a *SmMYB7*1-editing vector and a *SmMYB71*-overexpression vector, which were then delivered into the leaves of *S. miltiorrhiza* via *Agrobacterium*-mediated infiltration. This process ultimately yielded transgenic hairy roots of *S. miltiorrhiza*. The *rol*B and *HPT* genes were employed to identify the resulting hairy roots, and the ORF fragment containing the sgRNA was amplified and subjected to Sanger sequencing to ascertain the type of knockdown. In total, eight double-stranded *SmMYB71*-knockout lines and six *SmMYB71*-overexpression lines were successfully established.

**Figure 3:**
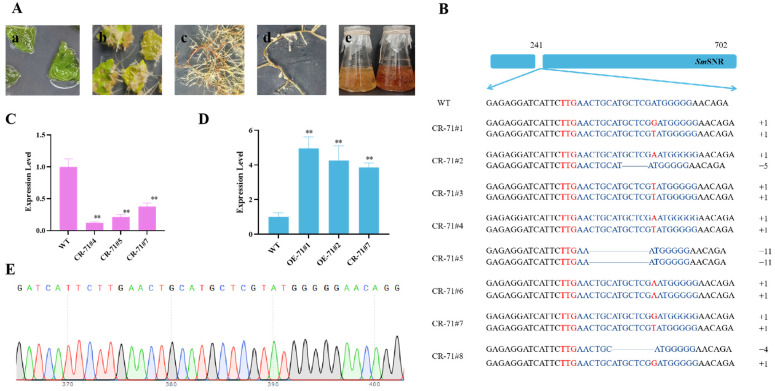
Identification of transgenic hairy roots. (**A**) Growth process of transgenic hairy roots. (**a**) Agrobacterium-infected leaves were placed flat on 1/2 MS medium. (**b**) Formation of healing tissue and rooting of hairy roots. (**c**) Hairy roots continued to grow (** p < 0.01). (**d**) Isolation of monoclonal hairy roots. (**e**) Culture for the expansion of hairy roots. (**B**) Mutations in *SmMYB71* mediated by the CRISPR/Cas9 system, along with the structure and editing site of *SmMYB71*. In the image, the editing sites are indicated in blue font, the first three nucleotides of the editing sites are shown in red font, and the base deletions are marked with solid lines. (**C**) Expression of the *SmMYB71* gene in knockout strains. (**D**) Expression of the *SmMYB71* gene in overexpression lines. (**E**) Representative mutations in *SmMYB71* induced by CRISPR/Cas9.

**Figure 4:**
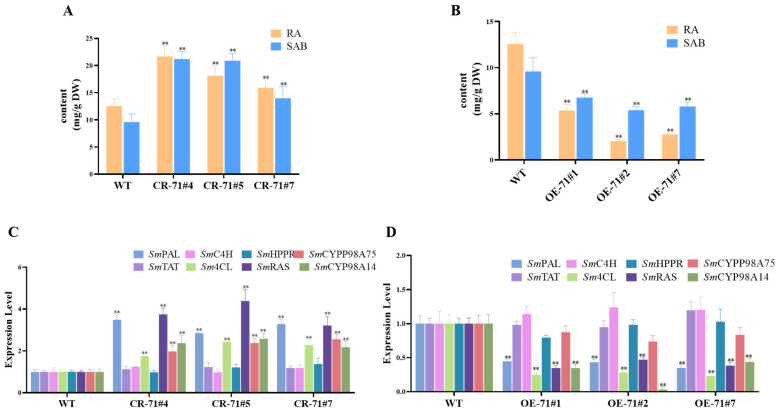
(**A**) Salvianolic acid content in hairy roots with *SmMYB71* knockout. (**B**) Salvianolic acid content in hairy roots with *SmMYB71* overexpression. (**C**) Expression levels of key enzyme genes in hairy roots with *SmMYB71* knockout. (**D**) Expression levels of key enzyme genes in hairy roots with *SmMYB71* overexpression. Data are presented as means ± SD of three biological replicates. Statistical significance was determined by Student’s *t*-test (** *p* < 0.01).

The expression of the *SmMYB71* gene in transgenic hairy roots was assessed using qRT-PCR. From this analysis, three *SmMYB71*-edited hairy roots (CR-71#4, CR-71#5, CR-71#7) and three *SmMYB71*-overexpressed hairy roots (OE-71#1, OE-71#2, OE-71#7) exhibiting the most significant expression changes were selected (Figure 3). The transgenic hairy roots were then analyzed and quantified for salvianolic acid content using UHPLC-MS/MS. Compared to the wild-type lines, the content of salvianolic acid was significantly increased in the *SmMYB71*-edited hairy root lines. Notably, in the CR-71#5 line, with the level of SAB being 2 times higher than that of the wild type, and the content of RA also increasing from 12.58 mg/g to 21.68 mg/g (Figure 4A). In contrast, the salvianolic acid content in the overexpression lines decreased significantly, with the level of SAB being about half that of the wild type, and the content of RA reducing from the original 12.58 mg/g to 2.04 mg/g in OE-71#4 (Figure 4B).

### 2.5. Knockout of SmMYB71 Promotes the Expression of Key Enzymes in Salvianolic Acid Biosynthesis

To further investigate the impact of *Sm*MYB71 on key enzyme genes involved in salvianolic acid biosynthesis, relative expression analysis was conducted on *SmMYB71*-edited hairy roots and *SmMYB71*-overexpressing hairy roots. The results indicated that in the *SmMYB71*-overexpression lines, the expression levels of the salvianolic acid biosynthesis-related enzyme genes *SmPAL*, *Sm4CL*, *SmRAS*, and *SmCYP98A14* were significantly downregulated (Figure 4C). In contrast, after the knockout of the *SmMYB71* gene, the expression levels of the *SmPAL*, *Sm4CL*, *SmCYP98A75*, *SmCYP98A14*, and *SmRAS* genes all increased (Figure 4D).

### 2.6. SmERF115 Enhanced the Transcription Level of SmMYB71 by Binding to Its Promoter

**Figure 5:**
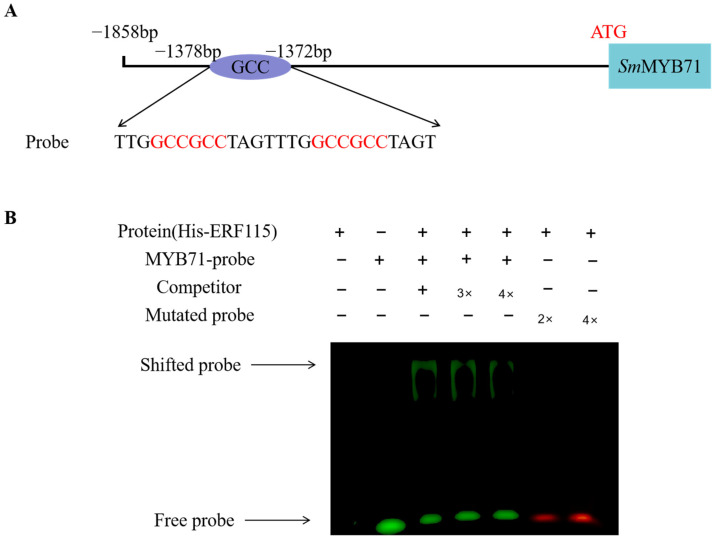
The regulatory effect of *Sm*ERF115 on the *SmMYB71* promoter is depicted in (**A**), which presents a schematic diagram of the *SmMYB71* promoter, highlighted in the GCC box in red font. (**B**) EMSA experiments were conducted using wild-type and mutant probe sequences. The wild-type probe, which contained the GCC box, was fluorescently labeled with HEX at the 5′ end, while the mutant probe was labeled with FAM at the same position. The symbols “+” and “−” indicate the presence and absence of the respective probes.

Previous studies have reported that AP2/ERF family proteins directly bind to the GCC binding site. The sequence of the *SmMYB71* promoter was analyzed, and a GCC motif was found located at 1378 bp upstream of *SmMYB71* the starting position. To verify *Sm*ERF115 binding to the promoter of *SmMYB71*, EMSA was performed with the His-*Sm*ERF115 proteins from prokaryotic expression using a specific *E. coli* strain. His-*Sm*ERF115 and a DNA probe including the GCC box were present, and the occurrence of a single shifted band was detected. As the cold competitor concentration increased, the intensity of the band decreased, suggesting *Sm*ERF115 was exclusively bound to the *SmMYB71* promoter’s GCC box (Figure 5B). The above results indicate that *Sm*ERF115 inhibited the expression of *SmMYB71* by directly binding to the promoter of *SmMYB71*.

## 3. Discussion

### 3.1. SmMYB71 Is a GA-Induced TF in S. miltiorrhiza

Phytohormones play critical roles in plant growth, development, stress responses, and secondary metabolism. For *S. miltiorrhiza*, the majority of phytohormone-related research focuses on the role of MeJA-related TFs in salvianolic acid biosynthesis, while investigations concerning GAs in salvianolic acid biosynthesis remain superficial. GAs are significant phytohormones that regulate plant development and reproduction; GA-induced genes are crucial for seed dormancy and germination, plant height, and biomass, as well as abiotic and biotic stress tolerance. For instance, the mutation of *LEA33*, one of five (*LEA* genes, promoted seed germination by enhancing GA biosynthesis in rice embryos [18]. In *Arabidopsis*, *DDF1* interacted with *AtGA2ox7* to upregulate gene expression, resulting in GA deficiency and improved salt tolerance [19]. However, few studies have concentrated on the role of GAs in plant metabolism.

In our study, *Sm*MYB71 was characterized from a transcriptome database of hairy roots treated with GAs. To confirm the fold change in the response of *SmMYB71* to GAs, qRT-PCR was employed to assess the expression levels in both hairy roots and *S. miltiorrhiza* plants under GA_3_ treatment. The results were consistent with the transcriptome database, showing a significant decrease in the expression level of *SmMYB71* following GA_3_ induction. The expression level of *SmMYB71* decreased dramatically at 3 h after induction and gradually returned to baseline at 6 h. This result demonstrated that *Sm*MYB71 functions as a GA-responsive TF in *S. miltiorrhiza*. The study regarded the response of MYB TFs to GA treatment in *S. miltiorrhiza* as limited, and it will reveal the role of GA-responsive MYB TFs.

### 3.2. SmMYB71 Negatively Regulates Salvianolic Acid Biosynthesis in S. miltiorrhiza

Several MYB TFs are involved in the regulation of GA metabolism. For example, *Cm*MYB2 may regulate flowering by inhibiting the activity of the *Cm*BBX24 protein and modulating the GA pathway in *Chrysanthemum morifolium* [20]. In *N. tabacum*, *Nt*MYB330 specifically regulates proanthocyanidin biosynthesis through the formation of the MBW complex in tobacco flowers and affects germination by adjusting proanthocyanidin concentrations and abscisic acid (ABA)/GA signaling in tobacco seeds [21]. Given that proanthocyanidins are important phenolic compounds, their regulatory network provides insights into the biosynthesis of salvianolic acid in conjunction with GAs and MYB TFs in *S. miltiorrhiza*.

In a previous study, the accumulation of salvianolic acids significantly increased in hairy roots of *S. miltiorrhiza* following GA treatment, with the key enzymes in the salvianolic acid biosynthesis pathway being upregulated. However, the specific mechanism behind this phenomenon remains unknown [10]. Based on the role of MYB TF in secondary metabolite regulation, we analyzed the expression levels of key enzymes involved in the salvianolic acid biosynthesis pathway using the transcriptome database of hairy roots treated with GA_3_. Our results showed that key enzymes such as *Sm*PAL, *Sm*4CL, *Sm*RAS, *Sm*CYP98A14, and *Sm*CYP98A75 were upregulated following GA_3_ induction. With a significant decrease in *SmMYB71* expression levels, we hypothesized that *Sm*MYB71 may play a crucial role in salvianolic acid biosynthesis. We constructed gene-knockout and overexpression vectors to investigate the function of *Sm*MYB71 using a hairy root system. This system is a convenient platform for studying gene functions because of its rapid growth, ease of obtainability, and minimal individual differences. Overexpressing *SmMYB71* in *S. miltiorrhiza* hairy roots significantly decreased the content of salvianolic acid via downregulating key enzymes in salvianolic acid biosynthesis. Knockout of *SmMYB71* resulted in higher salvianolic acid accumulation in transgenic hairy roots compared to wild-type (WT) lines, with the expression of key enzymes in the salvianolic acid biosynthesis pathway downregulated. These results suggest that *Sm*MYB71 functions as a TF that negatively regulates the accumulation of salvianolic acids.

### 3.3. SmMYB71 Is a Potential Target to Improve the Quality of S. miltiorrhiza

*S. miltiorrhiza*, belonging to the Labiatae family, is a notable medicinal plant. Its dried roots, known as *S. miltiorrhiza* in Chinese, have been extensively utilized in combination with other herbs for numerous years to address a variety of conditions, such as cardiovascular diseases, menstrual disorders, inflammation prevention, hepatocirrhosis, and as an anti-osteoporotic agent [2,22]. Various forms of clinical applications, including capsules, dripping pills, injection solutions, and tablets, incorporate *S. miltiorrhiza* [23]. Furthermore, as a medicinal herb with food-like properties, it has been shown to improve the quality of sleep in patients with insomnia when consumed as Danshen tea. Salvianolic acids, such as SAB and RA, exhibit potent antioxidant, anticoagulant, and anti-inflammatory properties [24,25]. In commercial *S. miltiorrhiza* decoctions, SAB serves as the predominant marker component utilized for quality control, as stipulated by the official Chinese Pharmacopoeia. These compounds have garnered growing attention in recent years, underscoring the necessity of enhancing the accumulation of salvianolic acids to elevate the quality of *S. miltiorrhiza* and meet market demands.

With the development of gene editing technology, the CRISPR/Cas9 system has rapidly garnered attention from both scientific researchers and industry professionals as a cutting-edge tool for genome and gene editing, particularly for enhancing crop breeding, with promising results. For example, Arginase (ARG) is an important enzyme regulating plant root development. Overexpression of ARG inhibited the formation of lateral roots [26]. Utilizing the CRISPR/Cas9 system to knock out the *ARG* gene in cotton can significantly increase the number of lateral roots in cotton (*Gossypium hirsutum*), promoting its growth and development as well as its response to external environmental stress [27]. In addition, by specifically knocking out two *BnaMAX1* homologous genes using the CRISPR/Cas9 system, the yield of rapeseed (*Brassica napus* L.) can be increased by 30% [28]. Although it has been a relatively short time since the natural genome editing tool was recognized, the CRISPR/Cas system has emerged as one of the most powerful tools in the history of molecular biology research. The development of CRISPR/Cas-based genome editing technology offers a novel opportunity for medicinal plant improvement through the use of this cutting-edge tool for precision breeding. However, identifying and selecting the desired genes associated with specific traits represents a key challenge in the precise enhancement of medicinal plant characteristics, particularly for traits with complex genetic backgrounds, such as yield and quality. In this study, we discovered a GA-induced TF *SmMYB71*, which negatively regulated salvianolic acid biosynthesis in *S. miltiorrhiza.* Knockout of *SmMYB71* increased the accumulation of salvianolic acid dramatically. This is a promising strategy to promote the quality of *S. miltiorrhiza* in metabolite engineering practice.

## 4. Methods and Materials

### 4.1. Plant Materials

The variety of *S. miltiorrhiza* used in this research was “*S. miltiorrhiza* f. alba”. Plants were cultivated using a soil-based method [peat moss: perlite, 2:1 (*v*/*v*)] in a greenhouse at 25 °C, with a photoperiod of 16 h of light and 8 h of darkness each day. The soil moisture content was maintained at 40–60%. The hairy roots were grown in 1/2 MS medium [MS (2.2 mg/L), sucrose (30 mg/L), agar (7 mg/L)], in dark conditions at 25 °C.

### 4.2. GA Treatment

Before the experiment, we separately used 50 μM, 100 μM, and 200 μM of GA_3_ to stimulate hairy roots of *S. miltiorrhiza*. The results showed that the expression level of the salvianolic acid biosynthesis pathway exhibited the best response to 100 μM of GA_3_. Therefore, we used 100 μM GA_3_ on two-month-old *S. miltiorrhiza* and hairy roots in the GA induction experiment. Before GA_3_ treatment, we collected samples from the roots of normally growing *S. miltiorrhiza*. After irrigating with GA_3_ for 3 h and 6 h, we took two additional samples. We also subjected the hairy roots of *S. miltiorrhiza* to the same treatment. Before the treatment, a portion of the hairy roots was collected, and then the roots were soaked in 100 μM GA_3_, followed by collecting another portion of the hairy roots for 3 h and 6 h.

### 4.3. Isolation and Characterization of SmMYB71

The RNA of *S. miltiorrhiza* was extracted using a TransZol UP Plus RNA Kit (Transgen, Beijing, China) and reverse-transcribed to obtain the cDNA of *S. miltiorrhiza* using One-Sept gDNA Removal and cDNA Synthcsis SuperMix (Transgen, Beijing, China). Amplification primers were designed using the primer designing tool (https://www.ncbi.nlm.nih.gov/tools/primer-blast/, accessed on 23 March 2023), to available on the NCBI website and the open reading frame (ORF) of *SmMYB71* was amplified by polymerase chain reaction using cDNA as a template using FastPfu Fly DNA Polymerase (Transgen, Beijing, China). The PCR conditions were pre-denaturation 95 °C for 2 min, 35 amplification cycles (denaturation 95 °C for 20 s, annealing 55 °C for 20 s, extension 72 °C for 1 min), and extension 72 °C for 5 min. PCR clones were recovered and ligated into Blunt-zero vectors and sequenced for comparison. The primers for the *SmMYB71* clones are shown in Table S1.

### 4.4. Phylogenetic Analysis of SmMYB71 Genes

The *SmMYB71* gene was analyzed using the BLAST tool P-BLAST (Protein BLAST: search protein databases using a protein query (nih.gov)) on the NCBI website to predict the structural domain of the gene. At the same time, we used this website to search and download the most similar genes to the *SmMYB71* gene in different species. The MEG 5.0 software was used to construct the phylogenetic evolutionary tree of this gene, and the sequence comparison was performed by MUSCLE; the Neighbor-Joining (NJ) method was used, default parameters were used, and the checking parameter Bootstrap was repeated 1000 times to construct the phylogenetic evolutionary tree.

### 4.5. Expression Analysis of SmMYB71

Roots, stems, leaves, and flowers of the same *S. miltiorrhiza* plant were collected and RNA was extracted using a TransZol UP Plus RNA Kit (Transgen, Beijing, China). Then, 1 μg of total RNA was reverse-transcribed into cDNA using One-Sept gDNA Removal and cDNA Synthcsis SuperMix (Transgen, Beijing, China). Quantitative real-time polymerase chain reaction (qRT-chain) was performed on a ROCHE LightCycler96 instrument using TB Green^®^ Premix Ex Taq TM (Tli RNaseH Plus) (Transgen, Beijing, China). The 18S ribosomal RNA gene was used as an internal reference primer for the quantitative polymerase chain reaction (qPCR). The qRT-PCR was performed on a ROCHE LightCycler96 instrument to detect the expression level of the *SmMYB71* gene in different organs of *S. miltiorrhiza*. The amplification conditions were pre-denaturation 94 °C for 30 s, 45 amplification cycles (94 °C for 5 s, 60 °C for 15 s, 72 °C for 10 s), and melting curve (95 °C for 15 s, 60 °C for 60 s, 97 °C for 1 s), and the primers used are shown in Table S1. The expression of the *SmMYB71* gene was calculated by the 2^−ΔΔCt^ method.

### 4.6. Subcellular Localization Assay

Specific primers containing *Xho*I and *Age*I enzyme cleavage sites (see Table S1 for primer sequences) were designed to amplify the complete ORF region of the *SmMYB71* gene (without terminator). And it was ligated into the pEAQ-GFP vector linearized with *Xho*I and *Age*I enzymes to construct a C-terminal fusion GFP protein. The constructed vector was transferred to *Agrobacterium tumefaciens* GV3101 and cultured in YEB medium containing kanamycin and rifampicin until OD_600_ = 0.8. Bacteria were collected by centrifugation and resuspended with transient transformation buffer (10 mM MgCl_2_, 10 mM MES, and 200 μM Acetosyringone). After standing at room temperature for 2 h, the upper portion of the more active bacterial solution was aspirated with a syringe and slowly injected into the abaxial surface of the *Nicotiana benthamiana* leaves. The injected *N. benthamiana* was left in the dark for 2 days. The tobacco leaves at the injection site were cut off and observed using a Leica TCS SP5 laser confocal scanning microscope (Leica Microsystems, Wetzlar, Germany) with an excitation wavelength of 488 nm and an emission wavelength of 520–548 nm.

### 4.7. Generation of SmMYB71 Overexpression and Editing of S. miltiorrhiza Hairy Roots

To construct the *SmMYB71*-editing vector, the ORF region of the *SmMYB71* gene was analyzed using the CHOPCHOP website (https://chopchop.cbu.uib.no/, accessed on 10 April 2023), to find potential sgRNA sequences, and *SmMYB71*-editing primers were designed and annealed. Linearization of psgR-Cas9-At was performed using the *Bbs*I cleavage site. The 20 bp DNA sequence formed after annealing was ligated into the linearized intermediate vector psgR-Cas9-At, which was driven by the AtU6 promoter. Then, the constructed CRISPR/Cas9 vector was cloned into the pCAMBIA1300 plant expression vector. For the overexpression vector, the PHB-myc vector was linearized using the *Hind* III and *Bam*H I enzymes, while the complete cDNA region of the *SmMYB71* gene was ligated into the linearized PHB-myc vector using homologous recombination, driven by a 35S strong promoter. The primers used in this experiment are shown in Table S1.

The constructed vector was transformed into *Agrobacterium rhizogenes* C58C1 and the transgenic hairy roots were obtained using leaf disk transformation. The transformed *Agrobacterium* C58C1 was utilized to infiltrate the leaves of *S. miltiorrhiza*, and at the end of the infiltration, the leaves of *S. miltiorrhiza* were laid flat on 1/2MS solid medium at 25 °C and dark-cultured for 2 days. At the end of the dark culture, the leaves were transferred to 1/2MS solid medium containing 500 mg/L of cephalosporin. The content of cephalosporin in the medium was then reduced every two weeks (250 mg/L, 200 mg/L, 150 mg/L). After the hairy roots grew to the appropriate size, genomic DNA of monoclonal hairy roots was extracted using the *EasyPure^®^* Plant Genomic DNA Kit (Transgen, Beijing, China) and positively verified using the primers in Table S1. Positive trichome roots were transferred to 1/2MS liquid medium containing 200 mg/L cephalexin to continue the expansion culture. The knockout hairy roots were also sequenced using the specific primers in Table S1 to further determine the knockout in the CRISPR/Cas9 transgenic hairy root.

### 4.8. Measured Content of RA and SAB in S. miltiorrhiza Hairy Roots

Salvianolic acid was extracted by taking 1 mg of grounded hairy root and sonicated with 75% ethanol. The content of RA and SAB in *SmMYB71*-knockout and -overexpression hairy root lines were determined using high-performance liquid chromatography–tandem mass spectrometry (UHPLC-MS/MS). The Nexera LC-40 chromatographic system (Shimadzu, Kyoto, Japan) was coupled to a triple quadrupole MS (LCMS-8060, Shimadzu, Kyoto, Japan). The general MS parameters were as follows: a nebulizer gas flow rate of 3 L/min, a drying gas flow rate of 10 L/min, a heated gas flow rate of 10 L/min, an interface unit called IonFocus (Shimadzu, Kyoto, Japan), a desolvation temperature of 526 °C, a desolvation line temperature of 250 °C, a heat block temperature of 400 °C, and a collision-induced dissociation gas pressure of 270 kPa. The column temperature was 30 °C, the flow rate was 0.3 mL/min, and the injection volume was 2 μL. The mobile phase consisted of water containing 0.1% formic acid (phase A) and acetonitrile containing 0.1% formic acid (phase B). The elution method was as follows: 0 min (5% B)/5 min (95% B)/7 min (95% B), with the final 3 min for post-run equilibration. The content calculation was performed using the standard curve. Accurately weighed SAB and RA standard substances were dissolved separately in chromatographic-grade methanol to prepare 1 mg/mL standard stock solutions. Then, 100 μL of each standard substance was taken, mixed, and diluted with chromatographic-grade methanol to prepare a mixed standard solution with a concentration of 5 μg/mL. Subsequently, a gradient dilution using chromatographic-grade methanol was performed at a ratio of 1:2 to prepare a total of 6 different concentrations of standard solutions for the construction of a standard curve.

### 4.9. Electrophoretic Mobility Shift Assays (EMSAs)

The ORF of *SmERF115* with the stop codon removed was constructed into the pET-28a vector containing the His tag for expression purification using the kit. Probe primers containing GCC Box sequences were designed and modified with HEX fluorescence at the 5′ end of the forward primer. Additionally, the 5′ end of the mutation probe’s forward primer was labeled with 6-FAM. The primer sequences are presented in Table S1. The experiments were conducted in accordance with the experimental protocol utilizing EMSA kits (Beyotime, Shanghai, China), and the results were analyzed using The Sapphire^TM^ Biomolecular Imager (Azure Biosystems, Dublin, CA, USA).

### 4.10. Statistical Analysis

All experiments were repeated at least three times and results were analyzed using GraphPad Prism to analyze differences between groups using unpaired *t*-tests and one-way analysis of variance (ANOVA).

## 5. Conclusions

In this study, we identified GA-induced *Sm*MYB71, which is the largest salvianolic acid biosynthesis regulatory factor in the plant TF family. *Sm*MYB71 is a nucleus-localized R2R3-MYB TF, with the highest expression levels observed in leaf tissues. The study found that the content of RA in the *SmMYB71*-knockout root hair lines was 1.5 times higher than that of the control group, while the content of SAB was twice that of the control group. In contrast, in the *SmMYB71*-overexpression lines, the levels of RA and SAB were only 30% and 60%, respectively, of those in the control group. These results indicate that *Sm*MYB71 negatively regulates GA-mediated phenolic acid biosynthesis in *S. miltiorrhiza*. Meanwhile, the knockout of the *SmMYB71* gene significantly upregulated the expression of genes involved in the salvianolic acid biosynthesis pathway, including *SmPAL*, *SmRAS*, *Sm4CL*, and *SmCYP98A14*. Conversely, overexpression of the *SmMYB71* gene reduced the expression levels of relevant genes in the salvianolic acid biosynthesis pathway.

**Figure 6:**
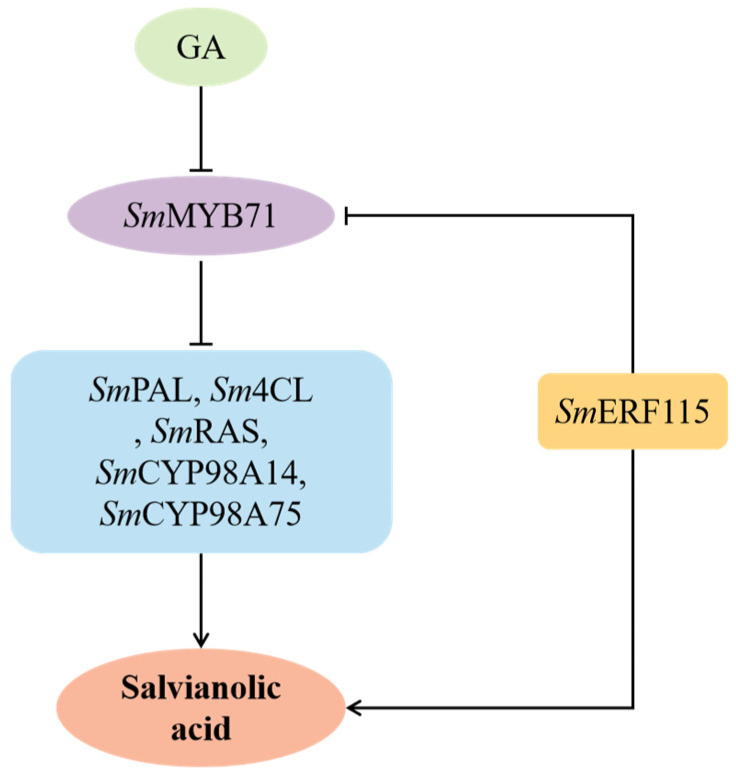
The regulatory model of salvianolic acid biosynthesis by *Sm*MYB71. GA negatively regulates the expression of *SmMYB71*. *Sm*ERF115 interacts with *Sm*MYB71 to inhibit its expression. Furthermore, *Sm*MYB71 inversely modulates the expression of associated genes (*SmPAL*, *Sm4CL*, *SmRAS*, *SmCYP98A14*, *SmCYP98A75*), thereby governing the biosynthesis of salvianolic acid.

In a previous study, the AP2/ERF TF *Sm*ERF115 from *S. miltiorrhiza* was characterized and found to be involved in the regulation of salvianolic acid biosynthesis. The production of salvianolic acid was increased in *SmERF115*-overexpressing hairy roots, while a decrease was observed in *SmERF115* RNA interference (RNAi) lines. EMSA demonstrated that *Sm*ERF115 directly bound to the promoter of *SmRAS* [29]. In the present study, we find that *Sm*ERF115 also binds to the promoter of *SmMYB71*. Furthermore, we elucidate a novel mechanism in which the *Sm*MYB71/*Sm*ERF115 module regulates the biosynthesis of salvianolic acid in *S. miltiorrhiza* (Figure 6).

## Data Availability

The data that supports the findings of this study are available in the Supplementary Material of this article.

## Supplementary Materials

The following supporting information can be downloaded at: https://www.mdpi.com/article/10.3390/molecules29245892/s1: The primer sequences used in the article.
