# Supplementary material for: Gibberellin-Induced Transcription Factor SmMYB71 Negatively Regulates Salvianolic Acid Biosynthesis in Salvia miltiorrhiza
Source: Molecules. 2024 Dec 13;29(24):5892. doi: 10.3390/molecules29245892 (PMC11679863; doi:10.3390/molecules29245892)
Supplement: Supplementary file 1 [file molecules-29-05892-s001.zip › molecules-3298445-supplementary.pdf]

Table S1

Primer	Purpose	Sequence (5'→3')
*Sm*MYB71-F	Gene Amplification	ATGTCTTGGGGTATGGGGTG
*Sm*MYB71-R		TGGTTCTCAATTAAGTAAGGGAAAT
*Sm*ERF115-F		CAATGGAAGCATCGGCGCA
*Sm*ERF115-R		TAATCATCCGTTTGGGTTATTCGG
*Sm*MYB71-qPCR-F	qPCR	CGAAGGCCGTTGGAACAATG
*Sm*MYB71-qPCR-R		CCATCTGTTCCCCCATCGAG
18S-F		ATGATAACTCGACGGATCGC
18S-R		CTTGGATGTGGTAGCCGTTT
*Sm*MYB71-Cas9-F	Vector construction and validation	GATTGTTGAACTGCATGCTCGATGG
*Sm*MYB71-Cas9-R		AAACCCATCGAGCATGCAGTTCAAC
PHB-*Sm*MYB71-F		accagtctctctctcaagcttATGTCTTGGGGTATGGGGTGG
PHB-*Sm*MYB71-R		gctcctgcagctcgaggatccATAAAAATCTAAATTTTGCTGGAAATTAG
*rol*B-F		CGAGGGGATCCGATTTGCTT
*rol*B-R		GACGCCCTCCTCGCCTTCCT
HPT-F		CGATTTGTGTACGCCCGACAGTC
HPT-R		CGATGTAGGAGGGCGTGGATATG
*Sm*MYB71-GFP-F	Subcellular localization	caaaaaagcaggcttctcgagATGTCTTGGGGTATGGGGTGG
*Sm*MYB71-GFP-R		cttgctcaccattccaccggtATAAAAATCTAAATTTTGCTGGAAATTAG
pET-*Sm*ERF115-F	Protein Expression	cagcaaatgggtcgcggatccATGGAAGCATCGGCGCAG
pET-*Sm*ERF115-R		ttgtcgacggagctcgaattcTCCGTTTGGGTTATTCGGATC
*Sm*MYB71-EMSA-F	EMSA	TTGGCCGCCTAGTTTGGCCGCCTAGT
*Sm*MYB71-EMSA-R		ACTAGGCGGCCAAACTAGGCGGCCAA
